# Cheoplasty

**Published:** 1859-07

**Authors:** James Moore


### ARTICLE V.

### *Cheoplasty.*

Messrs. Editors:

The following testimony of the worth of Cheoplasty is from the pen of a gentleman well known here at home for his estimable qualities; in offering it, he only expresses his own gratitude for the blessing he has enjoyed in its use, and a commendable desire that it may be known among all men.

A. J. B.

Baltimore, *May 27th*, 1859.

---

Baltimore, *May 26th*, 1859.

Dr. A. J. Brown,

*Dear Sir*:—In reply to your interrogatory respecting my present sentiments concerning "cheoplasty," I have only to say to you, what I have been called upon repeatedly to declare, in answer to communications from various parts of the United States, to wit: that up to the present hour, I have not found the slightest reason to change or modify the opinion I had the honor to communicate to you more than two years ago. My longer experience only confirms me in the belief, that with reference to all the essential qualities required in a metal for dental purposes, Cheoplasty is superior to all other compounds or metals, and in the course of time will probably supersede them.

Very truly,

Yours, &c.

JAMES MOORE.

---

